# Behavioral factors underlying energy consumption pattern: A cross-sectional study on industrial sector of Bangladesh

**DOI:** 10.1016/j.heliyon.2022.e11523

**Published:** 2022-11-16

**Authors:** Md. Shafiqul Islam, Md. Azaharul Islam Rayhan, Tanvir Hassan Majumder

**Affiliations:** a Department of Nuclear Engineering, University of Dhaka, Dhaka 1000, Bangladesh; b Bangladesh Central Bank, Dhaka 1000, Bangladesh; c Energy Institute, University of Dhaka, Dhaka 1000, Bangladesh

**Keywords:** Energy conservation, Industrial sector, Questionnaire survey, Behavioral construct, Logistic regression model

## Abstract

Energy consumption and wastage are increasing proportionally with the growth of the industrial sector of Bangladesh. This study explores the energy consumption behavior of industrial staff in the light of sociodemographic and behavioral aspects using questionnaire sourced survey data. Based on the feedback of 827 employees from the 20 types of industries, behavioral constructs are formed using exploratory factor analysis and reliability analysis. In addition, a descriptive statistical analysis and a logistic regression model are used to identify the impact of these constructs as well as sociodemographic variables on staff’s consumption behavior. Eight constructs were selected based on the different behavioral theories and energy culture model. They include personal consumption behavior, technology adoption norms, training and supervision, openness to change, technological ignorance, energy self-efficacy, engagement, and responsibility. Analysis of the data reveals that awareness building program through training is notably neglected in the industries of Bangladesh. Also, the staff do not differ in consumption behavior in regards to gender, level of education, age-group, and educational background. Adopting new technologies, increasing awareness through training, and improving responsibility can significantly ameliorate consumption behavior, whereas ignorance of technology adoption makes consumption behavior worse. This work can be considered as baseline data on the energy conservation pattern in the industrial sector of Bangladesh. The findings recommend developing policies, regulations, and guides to give equal importance to both energy efficiency and conservation programs for exploiting maximum energy saving opportunities.

## 1 Introduction

Bangladesh is showing promising growth in industrialization as a result of an emerging economy. It has achieved a 12.67% annual growth rate through strong export and remittance, and secured a place in the list of top countries of the world in the production of readymade garment (RMG), textile, leather, and ceramic products [1]. The industrial sector is the largest energy consumer which accounts for 51.80% of the total energy consumption in 2018 [2]. Moreover, the continuous economic growth creates more energy demand. Over the last few years, the severe power crisis compelled the government to enter into contractual agreements for high-cost temporary power solutions, such as rental and quick rental power, and small independent power producers on an emergency basis [3]. Therefore, the government of Bangladesh is trying to fulfill its energy demand by largely subsidizing the energy and power sector to keep the energy bill down. Industries are currently facing a 40 percent increment in their energy bills. To cope with the rise of production costs due to the increasing energy bills, energy experts emphasize energy efficiency [4]. Also, global warming is rapidly rising because of more energy generation and consumption, which should motivate our industries to embark on energy efficiency and conservation (EE&C) measures.

EE&C both measures aim for energy saving. Energy efficiency is related to the use of energy efficient appliances. But the inefficient use of energy efficient appliances makes us concerned about the expected amount of energy savings. That is where the concept of energy conservation exactly fits. Energy conservation involves the reduction of energy consumption through adjusting mindsets, behaviors, and habits. Energy conserving mindsets and habits also encourage the adoption of energy efficient technologies. Though adopting energy efficient technology can save a lot of energy, some problems also emerge. Firstly, after weighing the very high initial cost of purchasing energy-efficient appliances against the expected benefits of future cost savings, people get demotivated [5]. It is called “long pay-back periods” in the energy literature. Secondly, there are technical challenges (workforce and skill barriers) as well as infrastructural challenges (refurbishment of buildings) in adopting energy efficient appliances [6]. Lastly, political barriers and institutional shortcomings also play a negative role in adopting efficient technology. Therefore, the adoption of efficient technology should not be the only method of saving energy, energy conservation should also go hand in hand.

In the global context, researchers are working relentlessly to find out the scopes for the implementation of energy efficiency measures in industries. They are also investigating barriers and drivers of energy efficiency, as well as technological, and managerial solutions to minimize the energy wastage in industries. Thollander et al. [7] and Hasanbeigi et al. [8] independently investigated the barriers to the adoption of energy efficient measures and provided corrective measures to surmount those barriers. Researchers identified that adding insulation and building envelope [9], installation of energy efficient windows [10], cooling recovery [11], increasing walls and roof thickness [12], and scheduling of lighting and equipment [13] are found to be effective in energy conservation. Ngai et al. [14, 15] described a methodology and a framework for conserving energy by managing utility usage. Sawang et al. [16] and Zibarras et al. [17] concluded that office management and decision-making authority play an important role in energy conservation. Furthermore, increased technical monitoring, tracking daily energy consumption data within the workplace, and providing feedback using visualization, provide great potential for energy saving [18, 19, 20]. Arimura et al. [21] found that employees working in organizations with strict energy management systems are also likelier to practice energy conservation at home. From their studies, it is seen that researchers specially emphasize technology based solutions as well as proper energy management systems. Nevertheless, the adoption of highly expensive modern technologies is not popular enough to reduce energy consumption and to date, there are models based on behavior change theories that verify the possibilities of energy conservation. Stephenson et al. [22] offer a multi-disciplinary integrating model of energy behavior, a culture-based approach to assist in understanding the factors influencing energy consumption behavior. Zhang et al. [23] examined that the employee’s electricity consumption behavior is based on the norm activation model (NAM). There are several proposed models based on the theory of planned behavior (TPB). TPB is used to analyze the behavior pattern of an industrial worker in energy saving activities. Their findings suggest that the conservation of energy at the workplace is influenced by belief, intention, habit, and level of awareness, and there is a potential for saving energy through behavioral change [24, 25, 26, 27]. Minimizing the differences between the actual level of energy performance and the application of technologies with organizational behavior change can encourage more energy conserving practices [28].

In the context of energy efficiency and conservation in the industrial sector of Bangladesh, Sarkar et al. [29] examined the green business performance of the RMG sector. Based on the findings, the authors proposed for implementation of green technologies, strict governmental rules, and tax rebates. Reza et al. [30] recommended adopting green building technologies, low interest green financing, lowering the price of green technologies, and governmental aid like incentives and facilitating eco-friendly industrial zone. Habib et al. [31] described the possibilities of reducing energy consumption and emission in the garment industry by implementing energy efficient technologies in motor driven systems and lighting. Haque [32] suggested an awareness program on energy efficiency, easing the process of accessing financial assistance and developing a national strategy for promoting the widespread adoption of energy efficiency measures. Hasan et al. [33, 34] investigated the barriers and drivers for energy efficiency in Bangladeshi textile mills and steel mills. They identified inadequate technical cost-effective measures, capital expenditure, and poor research as barriers; and technology adoption as the most important driver for the adoption of energy efficiency measures. Sultana et al. [35] suggested that investors prefer an industry with standard environmental practices for its eco-friendly production and energy management efficacies. All of these studies are mainly focused on improving policy, regulation, and government incentives for the adoption of energy efficient technologies. However, the energy conservation issue is not emphasized.

As of July 2022, due to the fuel crisis and the high prices caused by the Ukraine-Russia war, the government of Bangladesh is compelled to roll blackouts (loadshedding) in order to import less fossil fuel for generating power. Now, the necessity of energy conservation is seen as the topmost priority to mitigate the power crisis. The government of Bangladesh is trying to save around 1000–1500 MW of electricity by mandating consumption reduction through area-wise load-shedding, and energy conservation at all levels [36]. It indicates the magnitude of practicing energy conservation at an individual level by revealing the amount of energy saved daily at an aggregate level. There is a clear message for everyone that if we can reduce energy wastage by maintaining proper consumption behavior in our daily life, we need to produce less energy in this sector. But there are lack of proper policies, regulations, and practices to cultivate this magnificent habit among industrial staff and the general public. Eventually, none of the studies addressed energy conservation through behavior change in the industrial sector of Bangladesh. Their studies are mainly focused on energy saving opportunities through energy efficiency measures against a particular type of industry.

However, the amount of energy that can be conserved through behavior change is huge, even though it is heavily ignored among all stakeholders. Energy conserving mindset and attitudes heavily influence the energy efficiency indicators. Thus, human factor/performance is the de facto best route to grow energy culture for minimizing energy wastage. This paper aims to provide an empirical overview of the behavioral factors underlying energy conservation pattern in the industrial sector of Bangladesh using a questionnaire based survey. The study investigates the behavioral factors considering several constructs for mapping the existing energy use pattern among different cross-sections of industrial staff to identify any gaps for future research and policy directions.

The layout of the paper is divided into five sections. Section 1 narrates the background and objectives of this research discussing existing knowledge and research gaps, section 2 describes the methods and tools carried out in this research, section 3 expounds upon the results, section 4 discusses a comparative analysis concerning similar studies and finally, a conclusion is drawn in section 5 alongside some policy recommendations.

## 2 Methodology

### 2.1 Questionnaire preparation

A survey questionnaire was developed based on the review of the literature by an expert team to assess the energy use pattern of individuals working in the industrial sector of Bangladesh. The questionnaire had three distinct parts. In the first part, the sociodemographic and personal information of the industrial respondents were asked. The second part was related to the understanding of the energy consumption behavior and attitude of the respondents. This section included closed ended questions on responsible use of electrical appliances both in the home and the office, the role of an individual in energy saving, and typical energy usage habits in the winter and summer seasons. The third part was on the general perception of the respondents regarding energy efficiency and motivational aspects of the respondents regarding energy conservation. Questions were evaluated using categorical responses, ordinal responses (5-point Likert scale), and continuous variables. Two forms of unipolar coding schemes were used for the 5-point Likert scale. For assessing the level of agreement of respondents, the agreement Likert scale contained “Strongly Disagree to Strongly Agree”. Similarly, the frequency Likert scale included “Never to Very often” as an option. Primarily, about a hundred (100) questions were prepared by the research team consisting of experts from different disciplines (energy experts, sociologist, and statistician) on the selected topic. Initially, the questions were prepared in English. Based on the pilot survey, confusing, lengthy, and double barreled questions were removed and translated into the local language of Bengali for the convenience of respondents. Finally, 38 questions were selected which included 4 sociodemographic questions along with 34 Likert items.

### 2.2 Data collection

According to the Economic Census conducted by the Bangladesh Bureau of Statistics [37], the total number of persons engaged in the industrial sector is 5080380 in 63,010 industries. According to this census, there are 20 types of industries which include the manufacture of food products, manufacture of beverages, manufacture of tobacco products, manufacture of textiles, manufacture of readymade garments (RMG), manufacture of leather and related products, manufacture of paper and paper products, printing and publication, manufacture of basic chemicals, fertilizers and nitrogen compounds, manufacture of pharmaceuticals, medicinal chemical and botanical products, manufacture of rubber and plastic products, manufacture of other non-metallic mineral products, manufacture of basic iron and steel, manufacture of fabricated metal products, manufacture of computers and IT products, manufacture of electrical equipment, manufacture of metal furniture, ships, and boats building industry, power plants, and gas distribution companies. Here, the sampling approach aimed to find a group of representative of the target population and to get a diverse set of respondents. For this, a convenient non-probability sampling technique was used to obtain the sample size, n = 850. To conduct the survey from the 20 types of industries, questionnaires were manually sent to them. The survey was conducted in person. In this study, administrative, managerial, and production staff were included in the data collection process. Prior to data collection, the ethical consent of each participant was taken. The survey was administered by experienced surveyors and two research assistants.

### 2.3 Data analysis

In the beginning, incomplete responses were screened out by the research team, which settled upon 827 responses from 850 sets of collected data. All statistical analyses were done using SPSS v 25 and Microsoft Excel. Four sociodemographic variables i.e., gender, age-group, level of education, and educational background along with 8 behavioral constructs e.g., personal consumption behavior (PCB), technology adoption norms, training and supervision, technological ignorance, openness to change, energy self-efficacy, engagement, and responsibility are taken into consideration. The scoring procedure of the behavioral constructs is based on the mean of some related Likert items. For example, the PCB score of an individual is the mean of the Likert items corresponding to the PCB construct (see Table 1). The rest of the constructs are scored in this way. Since a 5-point Likert scale (1–5) is used in this study, the mean of multiple Likert scale items also lies within this range. To identify whether the constructs were correctly formed or not, the internal consistency was tested to examine the reliability of the multiple-item Likert scale response using Cronbach’s Alpha. Alpha value ranging from 0.7 to 0.9 is acceptable and data in this range is considered to have better internal consistency. The constructs were formed on the basis of these Alpha values. An exploratory factor analysis (EFA) was conducted on the selected items using SPSS. EFA of the Likert items is useful to identify whether collectively the items represent one factor or more. It is used to increase the reliability of the scale by identifying and removing inappropriate items. In this study, the principal components method is used for factor analysis, and components having an Eigen value larger than 1.5 are not considered for further varimax rotation. Statistical tools like pie diagram, bar diagram, ANOVA test with means plot, and Kendal’s tau-b correlation test are employed to describe the sociodemographic variables, Likert items, and formed constructs. Here, personal consumption behavior (PCB) is considered the dependent/response variable. The four sociodemographic variables and the other seven constructs are considered covariates or explanatory variables.

**Table 1:** Results of EFA for PCB along with Cronbach’s alpha, factor loadings, Eigen value and percentage of variation.

Personal consumption behavior	Loadings	Communalities	Cronbach’s alpha (α)	Eigen value	% variation explained
Switch off lights in a hallway or restroom when not needed.	.234	.451			
Wear informal and lighter clothes during summer season.	.668	.708			
Wear formal and additional clothes during winter season.	.701	.542			
Shut off computer monitors when not needed.	.535	.731	.727	2.724	38.914
Set computer power to ‘save mode’ to minimize power.	.480	.692			
Keeping doors and windows closed when AC is on.	.678	.545			
Use shades and blinds to control direct sun through windows in both summer and winter to prevent or encourage heat gain.	.585	.640			

Here, the mentioned behavioral factors were constructed after studying a wide range of behavior change theories and energy culture framework. It was also inevitable to modify the items of the behavioral factors to represent the individual’s respective behavioral perception according to the context of Bangladesh. The most widely used behavior change theories include the Theory of Planned Behavior (TPB), Norm Activation Model (NAM), and the Stephenson energy culture model. Ajzen’s [38] TPB postulates that behavior is influenced by intention which in turn is influenced by attitude toward behavior, subjective norm (an individual’s perception of the prevailing opinion of others), and perceived behavioral control (an individual’s perception of his or her capabilities of performing the behavior). Schwartz’s NAM [39] was developed to explain pro-social and altruistic behavior. The degree to which people feel morally obliged to adopt pro-environmental behaviors (personal norms) is believed to be determined by the extent to which they are aware of the environmental consequences attached to their behavioral choices (awareness of consequences) and by the extent to which they assume responsibility for these problems (ascription of responsibility).

To identify the potential factors affecting PCB, the logistic regression model is applied. Explanatory variables are included by observing the bivariate relationship structure of the covariates with the dependent variable. The variables which have a significant bivariate relationship with the response variable are included in the logistic regression model. To identify the relationship between a categorical variable and the response, an ANOVA test was performed. Again to find the relationship between the response and a construct, Kendal’s tau-b correlation coefficient is used. A logistic model is used to provide the probability of occurring 1 (usually the category of interest for the response) for a particular covariate after adjusting the other covariates.

**Figure 1:**
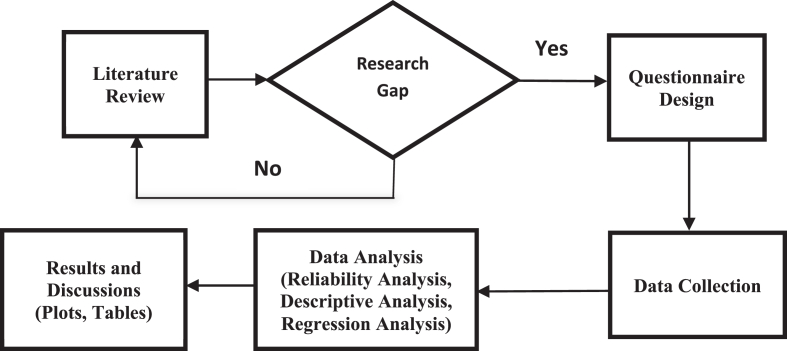
Research process.

According to Figure 1, we prepared a customized questionnaire for the industrial sector, collected data using this questionnaire, then finally cleaned and entered the data into SPSS using appropriate coding and scaling. Afterwards, we constructed behavioral factors using reliability analysis and exploratory factor analysis; graphically explored different aspects of the industrial sector; and analyzed the individual energy saving behavior using regression analysis.

## 3 Results

### 3.1 Reliability analysis

#### 3.1.1 EFA and Cronbach’s alpha

Personal Consumption Behavior (PCB) is the main construct of interest in this study and the items under this construct are displayed in Table 1. PCB indicates the perception of the respondents regarding their energy consumption habits and established energy use norms. A higher score of this construct for a respondent indicates his/her stronger belief regarding the practice of good and responsible energy consumption. Simultaneously, a higher score also means that respondents are practicing established practices in their industrial institution to reduce energy wastage. It is an acceptable construct (α = .727) and explains a 38.914% variation of the latent factor with an Eigen value of 2.724.

It is reported in Tables 2 and 3 that Technology Adoption Norms (TAN) represent the belief of the respondents regarding the readiness of their industrial institution to adopt new energy efficient technologies. A higher score for this construct indicated a perception that the industrial institution corresponding to the respondent was quicker to adopt new technologies and vice-versa. It consisted of 4 items and the Cronbach’s alpha score was acceptable (α = .726). Also, this construct satisfies the unidimensionality property because it explains 55.83% of the total variance of the latent factor alone according to EFA.

**Table 2:** Results of EFA. (TAN – Technology Adoption Norms, TS – Training and Supervision, TI –Technological Ignorance, OC – Openness to Change, ESE – Energy Self-Efficacy, Eng-Engagement, Res – Responsibility).

Question phrasing	TAN	TS	TI	OC	ESE	Eng	Res	Communalities
Employ a lower wattage of lighting where possible	.756							.571
Use automatic switches for lights, fans and AC to keep them...	.730							.533
Turn off all unnecessary lights	.802							.643
Minimize the heat created by lights, machinery	.698							.487
Authority organizes training to encourage and support staff...		.609						.370
Maintenance schedules include reducing energy wastage		.661						.437
I talk about saving energy with colleagues in the office.		.683		.634		.678		.466
In our meetings we discuss about energy efficiency.		.641		.697				.410
Instructing/encouraging/advocating colleagues to adopt energy		.687					.660	.472
Employees are trained properly to interact with any energy management...		.607			.675			.368
I don’t use energy efficient products too often.			.755					.658
Energy efficiency products do not always save energy			.772					.625
Lifecycle of products is too short			.762					.606
I have moral obligation to reduce my energy usage.				.530				.825
Taking action to reduce energy use is convenient for me				.554				.640
I attend regular management meetings to review energy use.				.711		.657	.695	.675
I consider energy management responsibilities as a part of...				.571			.539	.539
There should be an energy team to educate employees and operators...				.458				.621
I know nothing about energy efficiency labeling.			.665					.442
Taking action to reduce energy use is convenient for me					.844			.712
My action to reduce energy use is effective					.872			.761
I regularly watch documentary program regarding energy consumption issue in the television/internet.						.840		.868
I read lots of articles regarding energy consumption from the book/Magazine/Newspaper.						.794		.882
Actively report about energy wastage and give suggestions…							.691	.477
When leaving office, arrange for the last-man-out to check and switch off the power source to all air conditioning, lighting…							.616	.379
Switch off lights in a hallway or restroom when not needed.							.557	.310
Shut off computer monitors when not needed.							.593	.352

Extraction Method: Principal Component Analysis. Rotation Method: Varimax with Kaiser Normalization.

**Table 3:** Cronbach’s alpha of the newly formed constructs with Eigen value and percentage of variation explained.

Constructs	No. of items	Cronbach’s alpha (α)	Eigen value	% variation explained
Technology Adoption Norms	4	.726	2.233	55.830
Training & Supervision	6	.723	2.525	42.086
Technological Ignorance	4	.721	2.188	54.708
Openness to Change	7	.700	2.517	35.956
Energy Self-Efficacy	3	.715	1.929	64.293
Engagement	4	.728	2.226	55.647
Responsibility	7	.737	2.728	38.975

Table 2 outlines how Likert items from the survey map to the rest of the behavioral constructs. Table 3 provides all factor Eigen values and percentage variance explained along with internal consistency criteria mentioned in the previous section. Training and Supervision (TS) indicates respondents' perception regarding the availability of proper training facilities as well as the level of supervision in their respective industrial institutions. A higher score represents a stronger belief from the respondent regarding the existence of a proper energy management training and supervision system. It consisted of 6 items and the Cronbach’s alpha score was acceptable (α = .723). Here, this construct explains 42.08% of the total variation of the latent factor alone and all the factor loadings are greater than 0.5. Technological Ignorance (TI) is an inverse measure of technology related knowledge possessed by the respondent based on their beliefs. A higher score represents a higher level of belief regarding this type of ignorance and vice versa. It is an acceptable construct (α = .721) made of 4 items and it explains 54.708% variation in the latent factor according to EFA.

Openness to Change (OC) indicates the intention of the respondents to welcome behavioral change related to energy consumption in their respective industrial institutions. Higher scores indicated a stronger intention for accepting new changes in energy consumption behavior and vice versa. This construct is made of 7 items and it is acceptable (α = .700) according to Cronbach’s alpha score. Moreover, in EFA, OC explains 35.956% variation in the latent factor.

Energy Self-Efficacy (ESE) represents how participants feel about their ability to influence their energy use, concerning how easy it would be for their industrial institution to do so. Higher scores indicate that an individual feels it is easy for them to reduce their energy consumption. This is also an acceptable construct (α = .721) according to the measure of internal consistency (Cronbach’s alpha) and it consists of 4 items along with 54.708% explained variation in the latent factor according to EFA.

Engagement (Eng) is the construct to measure the level of active participation on the part of the respondent to reduce their energy consumption. A higher score of this construct indicates that an individual feels strongly positive about his/her role in reducing energy consumption in their respective organization. It is the collection of 4 items with an accepted score (0.728) in Cronbach’s alpha along with 55.64% explained variation in the unobserved factor according to EFA.

Responsibility (Res) represents the perception of the respondents about their duties and obligations toward their institution regarding the reduction in energy consumption as well as energy wastage. A higher score indicates a higher level of adherence to the belief that the respondent is responsible for his/her institution and vice versa.

### 3.2 Descriptive analysis

#### 3.2.1 Analysis on sociodemographic variables

**Figure 2:**
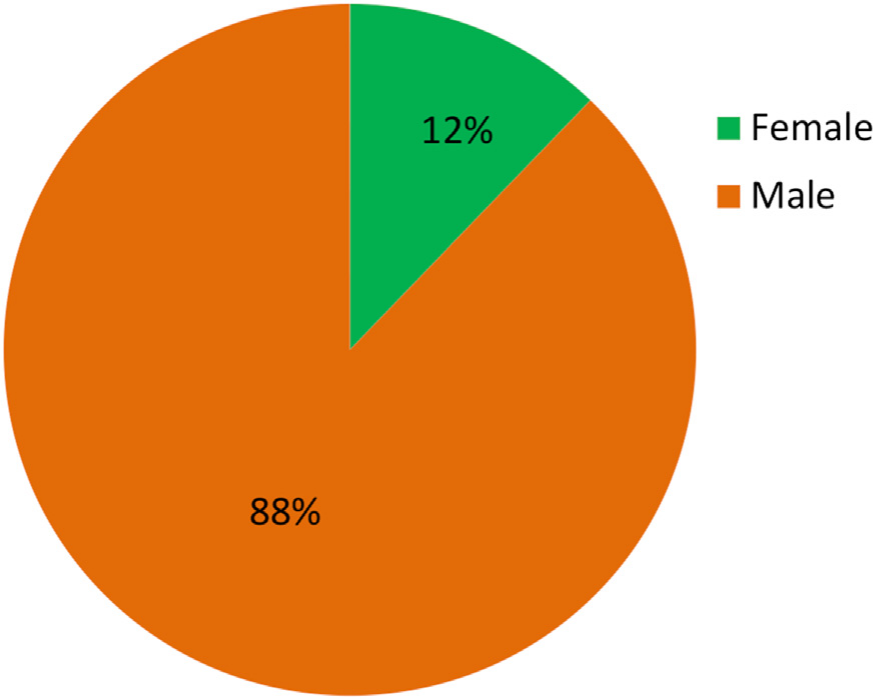
Gender distribution.

**Figure 3:**
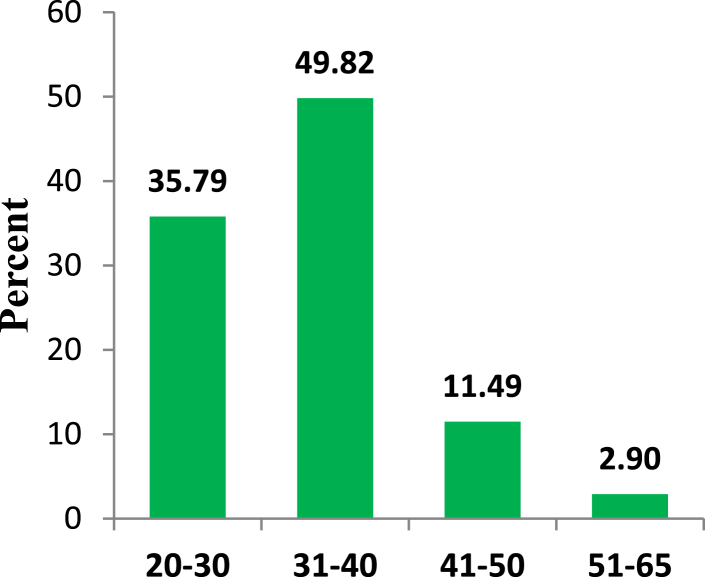
Age group distribution.

To describe the independent variable “Gender”, a pie diagram is shown in Figure 2. It is shown in Figure 2 that 88% of the respondents in the study were male. Figure 3 shows the maximum number of respondents who participated in this study from the industrial sector were aged between 31 and 40 years and they constitute almost 50% of our sample space. About 36% of the respondents were aged between 20 and 30 years.

**Figure 4:**
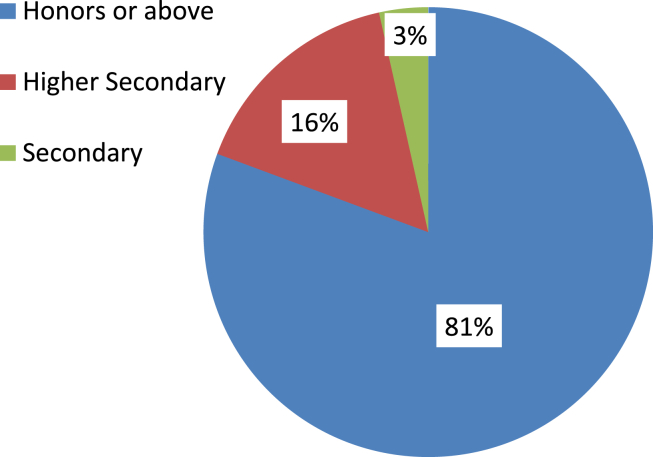
Level of education.

**Figure 5:**
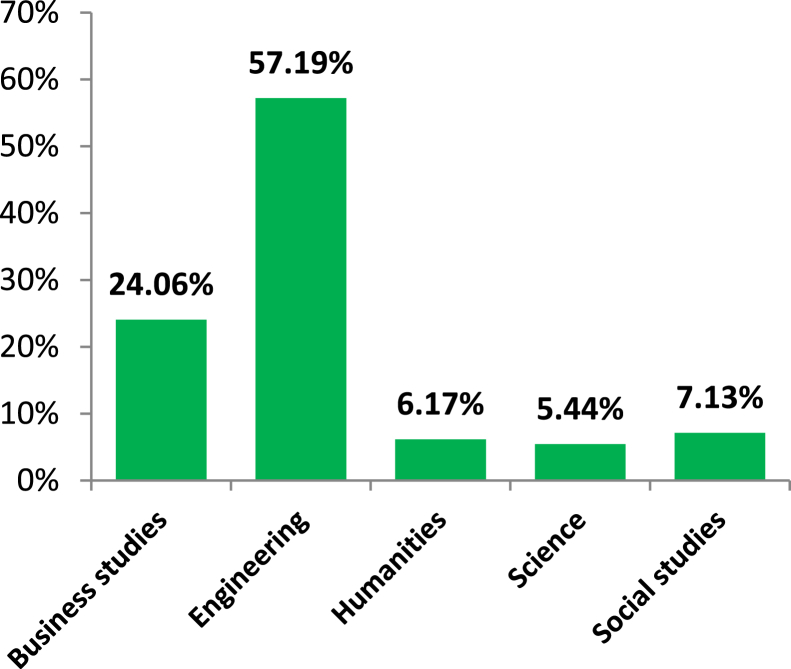
Educational background.

It is observed in Figure 4 that almost 80% of respondents hold at least an Bachelor’s degree and the rest of the respondents were either higher secondary or secondary school graduates. It is reported in Figure 5 that 473 respondents out of 827 were engineering graduates and they comprised about 58% of the total sample space. Also, around 24% of the respondents were from a business background.

**Figure 6:**
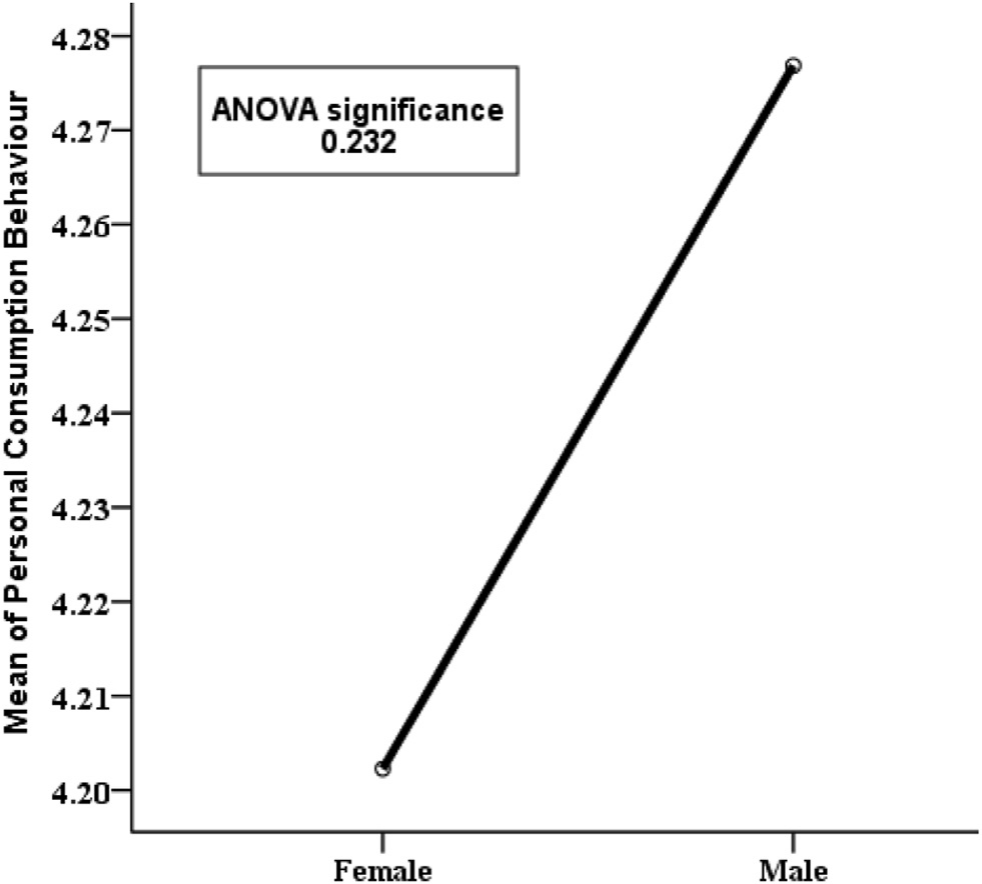
Variation in mean PCB values for categories of “gender’’ along with p-value of ANOVA test.

**Figure 7:**
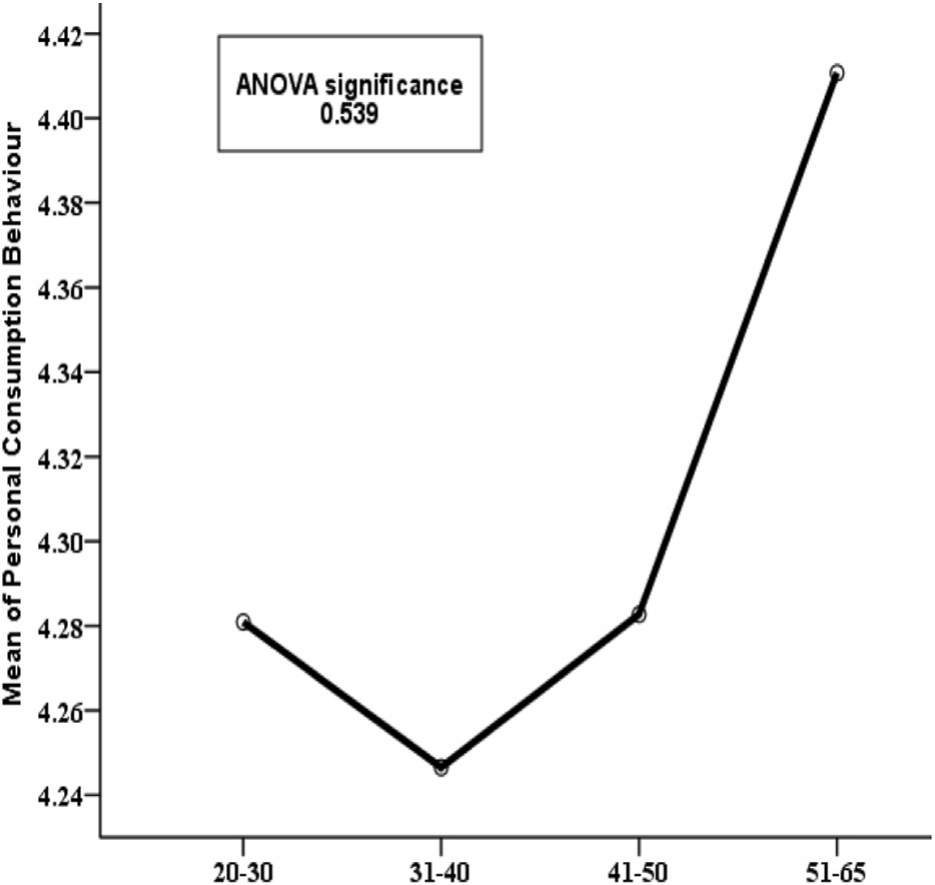
Variation in mean PCB values for categories of “age-group’’ along with p-value of ANOVA test.

**Figure 8:**
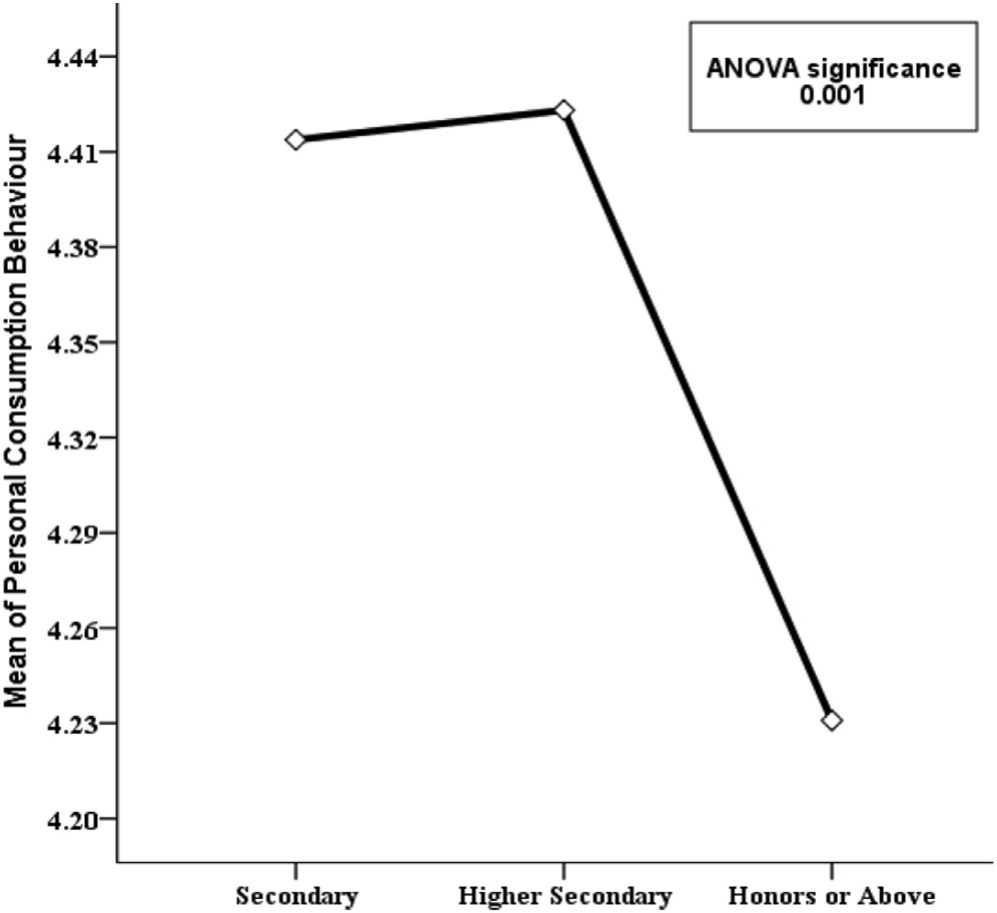
Variation in mean PCB values for categories of “level of education’’ along with p-value of ANOVA test.

**Figure 9:**
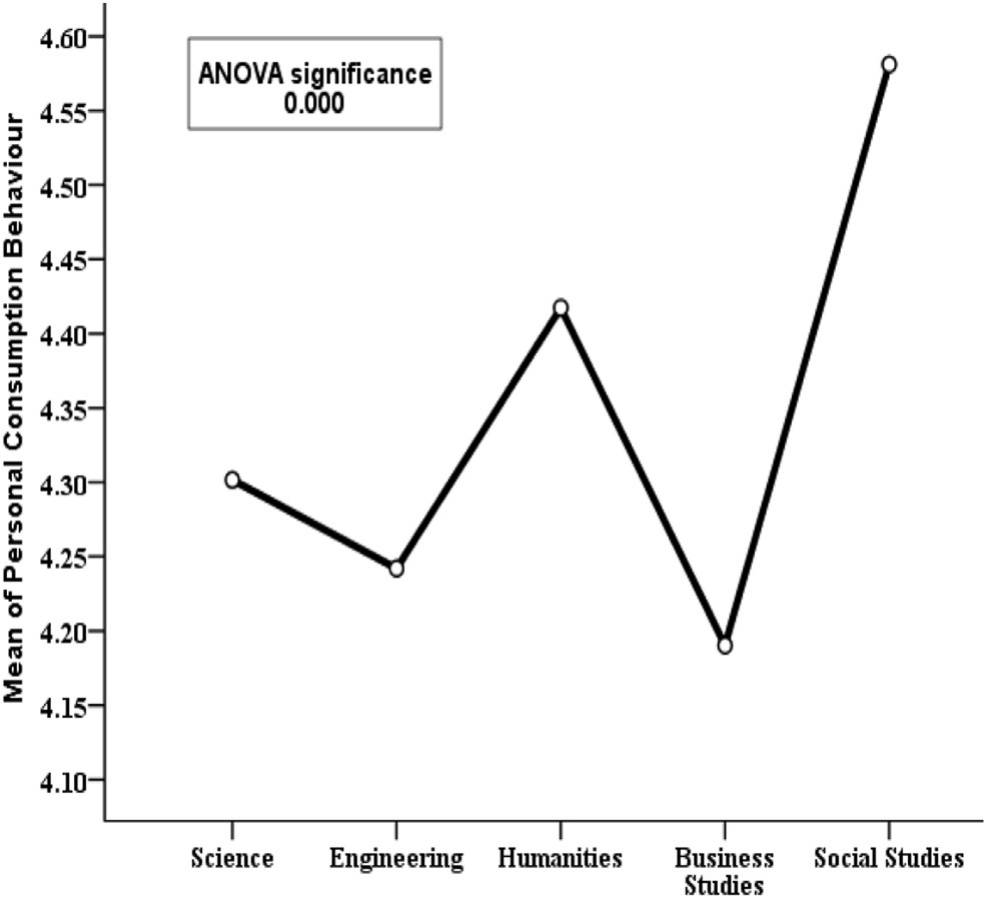
Variation in mean PCB values for categories of “educational background’’ along with p-value of ANOVA test.

For the purpose of describing PCB with respect to sociodemographic variables, an ANOVA test was performed and the results are portrayed in Figures 6, 7, 8, and 9. In these figures, the categorical sociodemographic variables are placed on the x-axis; and the mean PCB scores of the respondents who fall in the respective categories of the variable are placed on the y-axis. For example, according to Figure 6, the mean PCB is 4.20 for females and 4.28 for males. Here, the “mean PCB” score equal to 4.20 for female, means that the PCB score of each female respondent in this study is first summed and then averaged by dividing by the number of female respondents, which calculates to 4.20. Now, this slight difference between the male respondents and the female respondents with respect to the mean PCB score is not statistically significant as the p-value is 0.232. So, gender does not significantly impact PCB.

In Figure 7, the variable age group is represented by showing the mean PCB. It is found that respondents aged 51–65 years have a higher mean personal consumption score (4.41) compared to the other age groups. So, it seems like older people do more energy conservation than younger people but this finding is not statistically significant (p-value 0.539). Hence it can be concluded that age group also does not significantly affect PCB. Again, Figure 8 shows the mean PCB scores for categories of the variable “level of education”. It is clear from the diagram that respondents with secondary and higher secondary level education are pretty much similar (e.g., 4.41 & 4.42) in case of consumption behavior but those with at least Honors level of education have lower mean PCB scores. This indicates that secondary school and higher secondary level respondents show a more conserving nature compared to respondents with higher education. This finding is highly statistically significant as the p-value is 0.001. Similarly, it can be observed in Figure 9 that respondents with social studies and humanities backgrounds are showing more conserving behavior than those from other disciplines. Respondents from science, business, and engineering background have mean PCB scores of 4.30, 4.25, and 4.20 respectively, suggesting the similarity in consumption behavior. The differences in mean PCB scores between the above mentioned two groups are highly statistically significant as the p-value is 0.000. Hence, it can be concluded according to the ANOVA test that level of education and educational background are two significant sociodemographic variables. Thus, they are included in the logistic regression model to identify whether this finding stands still when the effects of the other variables are adjusted. Since one of our aims is to identify behavioral factors affecting PCB; the bivariate relationship of the behavioral constructs with PCB should be inspected along with the sociodemographic variables.

### 3.2.2 Analysis on selected Likert items and Kendal’s tau-b of behavioral constructs.

**Figure 10:**
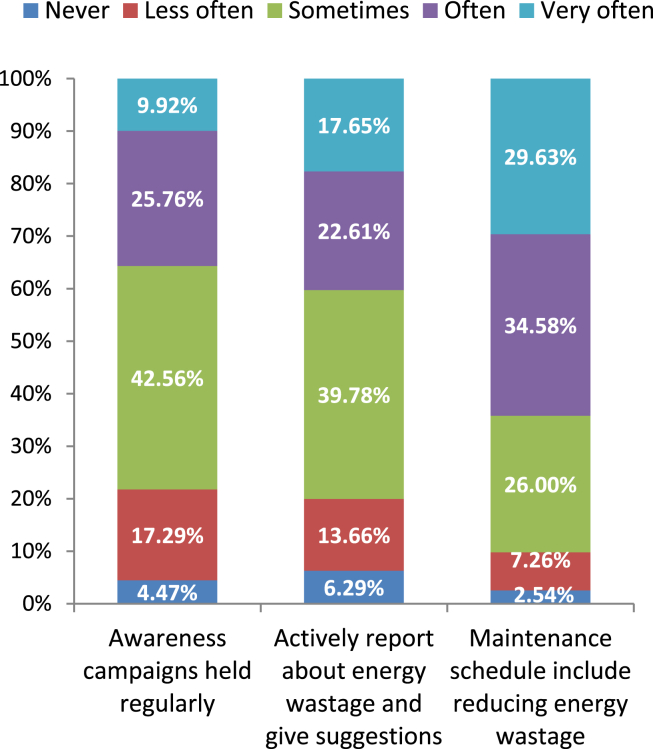
Perception on energy saving activities from management side and personal responsibility.

In this study, the total number of Likert items used to create the constructs is 31, and the total number of Likert items presented in the questionnaire is 34. To paint a picture of the industrial sector from the perspective of energy efficiency behavior, some Likert items are described here. Figure 10 shows the overview of the respondents regarding the regularity of awareness campaigns on energy efficiency. It can be observed that about 43% of respondents said awareness campaigns occur “sometimes” and about 18% said campaigns are arranged “less often”. This is an indication that the industrial sector is indifferent to energy conservation**.** Again, according to Figure 10, only about 30% answered “very often” to the statement regarding the inclusion of energy wastage reduction in the maintenance schedule. “Often” and “Sometimes” responses were provided by 35% and 26% of respondents respectively. So, it can be visualized from these three bar diagrams that the industries of Bangladesh are not taking energy wastage seriously and are giving less importance to energy efficiency measures. The level of support for the Likert item corresponding to whether respondents are active in reporting energy wastage and providing necessary suggestions to the institution is shown in Figure 10. Around 40% of respondents replied “sometimes” and only about 18% replied “very often”. This is obvious in the context of Bangladesh because the previous statements revealed the indifferent nature of the industries to energy efficiency measures. It is a fact when the authority is not serious about energy wastage, employees are more likely not to be.

**Figure 11:**
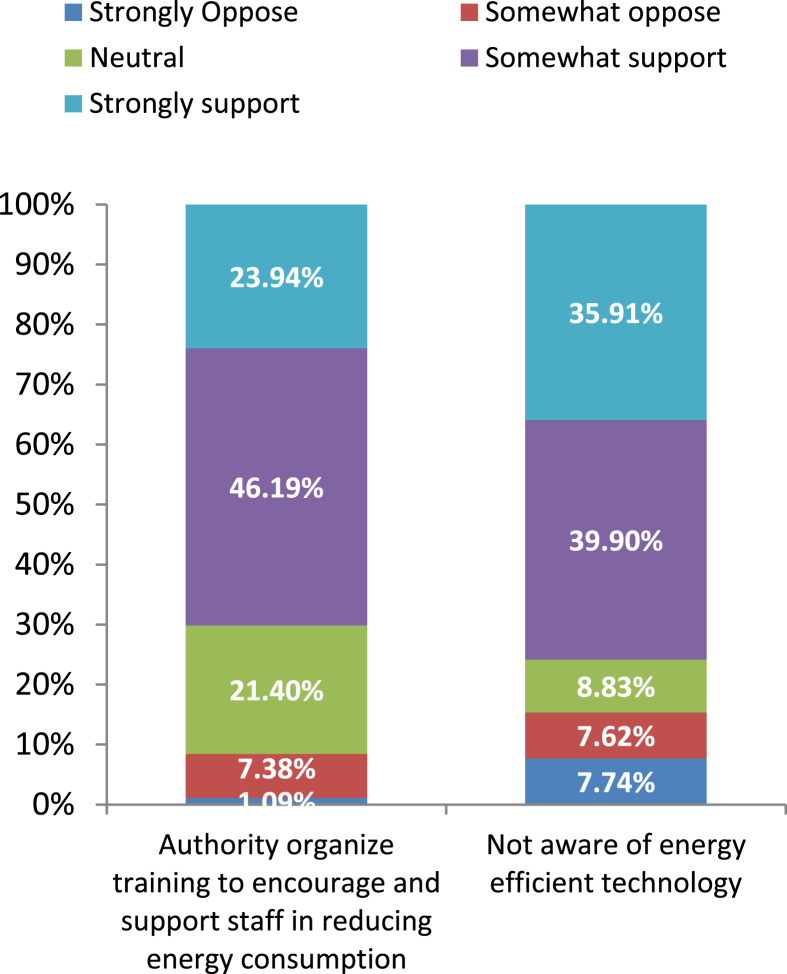
Feedback on training programs and personal unawareness.

Again, it is shown in Figure 11 that around 47% of respondents slightly support the fact that authority organizes training and around 22% of respondents remained neutral. Only about 24% of respondents strongly supported the statement, and this reflects the scarcity of energy conservation measures in the industrial sector of Bangladesh.

**Figure 12:**
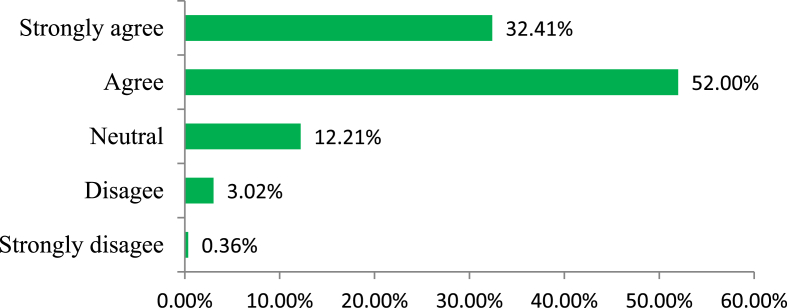
Feedback on the Likert item “Behavioral changes in energy use are important in my office”.

As a consequence of attending very few training sessions and awareness campaigns, respondents are not aware of the energy efficient technology (Figure 11). Figure 11 shows that about 76% of respondents have replied that they either somewhat or strongly support the statement regarding their lack of knowledge regarding energy efficient technology. It is evident in ​Figure 12 ​that behavioral change in the industries can play a major role to save energy.

Table 4 represents the non-parametric correlation coefficients (Kendal’s tau-b) between PCB and the constructs. It is reported in Table 4 that all the constructs have significant correlations with PCB because all the p-values are less than 0.05. Here, OC, Eff, and Eng have a moderately positive correlation with PCB but TAN has a mildly positive correlation. Training and supervision (TS) has a moderately positive correlation (0.415) with PCB. This means an increase in the score of TS that increases the score of PCB, indicating that better TS may lead to reduced energy consumption. Similarly, OC and Res are found to have a moderately positive correlation with PCB. However, the coefficient is highest for Res which means an improvement in the responsibility score. This may improve consumption behavior by reducing energy consumption. TI shows a weakly negative correlation with PCB. This reflects the fact that an increase in the score of this construct slowly decreases the PCB score. Since all the coefficients are statistically significant, all of them are included in the logistic regression model to identify their impacts after adjusting the other confounding variables.

**Table 4:** Kendal’s coefficient of correlations between PCB and each of the constructs.

Personal Consumption Behavior	Correlation Coefficient (Kendal’s tau-b)	Sig. (2-tailed)
Technology Adoption Norms (TAN)	.169∗∗	.000
Training and Supervision (TS)	**.415∗∗**	.000
Technological Ignorance (TI)	**-.114∗∗**	.000
Openness to Change (OC)	**.401∗∗**	.000
Efficacy (Eff)	.308∗∗	.000
Engagement (Eng)	.353∗∗	.000
Responsibility (Res)	**.521∗∗**	.000

### 3.3 Logistic regression model

For executing the logistic regression model, the dependent variable of PCB is divided into two categories. The PCB score is the mean of some Likert items. Values from 1 to 3.67 are coded as “others” and above 3.67 are coded as “green”. This classification based on a score is adapted from the technical guidance on “self-assessment of nuclear security culture in facilities and activities that use nuclear and/or radioactive material” by the international atomic energy agency (IAEA) [40]. Here, the “green” category indicates better energy conserving behavior and “others” means poor energy conserving behavior according to the definition of PCB. Numerically, “green” is given as code 1 and “others” is given as code 0 for computational purposes. It is assumed that there exists no interaction among the covariates of the model based on the collinearity diagnostics which is evident in Table 5. In Table 5, it is clearly shown that no variable surpassed the variance inflation factor (VIF) = 6 or 7 thresholds [41], and hence, the covariates included in the logistic regression model can be safely assumed to be free from collinearity problem.

**Table 5:** Collinearity diagnostics among the covariates of the logistic regression model using tolerance and variance inflation factor (VIF).

Characteristics	Tolerance	VIF	Characteristics	Tolerance	VIF
Higher Secondary	.190	5.269	Training and Supervision	.228	4.390
Honors or above	.170	5.892	Openness to change	.277	3.608
Engineering	.187	5.338	Technological Ignorance	.911	1.098
Humanities	.481	2.077	Efficacy	.533	1.876
Business Studies	.226	4.421	Engagement	.317	3.156
Social Studies	.443	2.260	Responsibility	.280	3.566
Technology Adoption Norms	.821	1.218			

The coefficients that came out as statistically significant in this model include Technology Adoption Norms (TAN), Training and Supervision (TS), Technological Ignorance (TI), and Responsibility (RES). It is found in Table 6 that an individual who supports the statements of TAN strongly is more likely to achieve “green” consumption behavior, or in other words, efficiency in energy consumption. Again, the finding related to TS is a confirmation of the previous findings related to PCB which indicates that improvement in the perception of TS improves the PCB. Therefore, industries need to promote training and proper supervision of individual respondents. This finding is significant at a 10% level of significance while insignificant at a 5% level of significance. It is statistically evident according to this model that an increment in the TI score will reduce the probability of achieving “green” PCB compared to “others”. In other words, it can be said in Table 6 that respondents who are technologically ignorant are more likely to possess inefficient energy consumption behavior (lower PCB score). This statement supports the general perception about a person who is ignorant of a topic. Responsibility is a very important construct and it came out as highly significant in this model with a p-value is 0.000. Here, it can be clearly observed that a responsible industry worker is very highly likely to possess a “green” consumption behavior. It is also a very prominent and well-established finding that responsible behavior can reduce the amount of energy consumption (larger PCB score).

**Table 6:** Estimates of the coefficients of logistic regression model along with standard error (S.E.) and statistical significance with color code of PCB as dependent variable.

Characteristics	Estimated coefficients	S.E.	Wald	Df	Sig.	Exp(B)
Secondary	-	-	-	-	-	-
Higher Secondary	-17.391	6665.200	.000	1	.998	.000
Honors or above	-17.550	6665.200	.000	1	.998	.000
Science	-	-	-	-	-	-
Engineering	-.257	.813	.100	1	.752	.774
Humanities	.003	1.169	.000	1	.998	1.003
Business Studies	-.751	.824	.830	1	.362	.472
Social Studies	-1.711	1.038	2.729	1	.101	.181
**Technology Adoption Norms**	.730	.238	9.384	1	**.002**	**2.074**
**Training and Supervision**	.867	.502	2.984	1	**.084**	**2.381**
Openness to change	-.610	.562	1.179	1	.278	.543
**Technological Ignorance**	-.528	.217	5.926	1	**.015**	**.590**
Efficacy	-.158	.356	.196	1	.658	.854
Engagement	-.359	.362	.983	1	.322	.698
**Responsibility**	3.773	.574	43.259	1	**.000**	**43.503**
Constant	6.281	6665.201	.000	1	.999	534.460

∗Nagelkerke R^2^ 0.566.

## 4 Discussion

Our study is focused on how behavioral factors influence energy conservation practices in the industries of Bangladesh. Our findings are consistent with the study of Zhang et al. [23] where it was found that along with other factors, “ascription of responsibility” is an important determinant of personal norm. Zierler et al. [42] published a case study where the newly formed constructs are used in a multiple regression path analysis to detect interrelationships. Efficacy, goal flexibility, and benefits evaluation were the factors found to be significantly affecting employees' energy behavior in Zierler’s study. This finding is based only on one institution which is not generalizable. It should be noted that some differences in the findings can be attributed to the prevailing culture of the country or region where the experiment takes place. Lopes et al. [43] studied industrial staffs' energy saving behavior by combining constructs from the Theory of Planned Behavior (TPB) and Norm Activation Model (NAM). In their study, two constructs are found to be important in explaining staffs' energy saving behavior: “Intention to Act” and “Personal Norms”. The items of “Intention to Act” and “Personal Norms” are similar to “Engagement” and “Responsibility” constructs but only “Responsibility” came out as significant in our study. Their study also dealt with only one chemical industry and hence cannot be generalized to the industrial sector to any extent, whereas our study dealt with 20 types of industries.

In this study, the adoption of any particular behavior model is avoided for twofold reasons. Firstly, behavior change models like TPB, NAM or Stephenson energy culture model do not include socio-demographic factors. For this, they remain inadequate to explain a sufficient amount of variation in behavior change. Secondly, the behavior of individuals varies greatly with regard to their socio-demographic characteristics. If we want to assess consumption behavior using a rigid statistical model, we have to employ socio-demographic variables along with behavior factors in those models.

For example, in the Lynch et al. [44] study, it was found that the TPB model is approximately valid for the data (behavioral intentions are significantly explained by perceived behavioral control and attitude toward a behavior). But only a trivial amount of change in electricity use is explained (R^2^ = 0.04) by the intentions and perceived behavioral control variables (both are insignificant) whereas the logistic regression model in our study which incorporated socio-demographic variables along with behavior factors can explain 56.6% (R^2^ = 0.566) of the total variation in PCB.

The implementation, plans, and actions related to energy efficiency in the industrial sector of Bangladesh is much more focused than the energy conservation program. The activities under energy efficiency program include use of different categories energy saving lights by replacing energy consuming incandescent bulbs, low energy consuming electric motors, and inverter addition air conditioners and refrigerators. However, energy conservation activities like training, education, awareness, conducting energy audit, and fostering energy culture are heavily inadequate.

Haque [32] studied the literature and existing energy-related policies to identify new policy goals and implementation barriers in adopting industrial energy efficiency in Bangladesh and provided recommendations to overcome these barriers.

In our study, TAN, a lack of awareness and adequate training programs in the industrial sector of Bangladesh is found which is consistent with the findings of Haque. However, Haque explored the industrial energy efficiency landscape in Bangladesh using a literature review whereas our study has provided recommendations based on the findings from survey data.

Our study has two distinct specialties. Firstly, this study has used constructs that are not based on any specific behavior change model i.e., TPB, NAM, or Stephenson energy culture model; and secondly, data is obtained from 20 types of industries having various locations across the country using a questionnaire survey.

This study suggests that policymakers should focus on regulating the industries to arrange regular training, education, and awareness programs to educate and aware the industrial staff on energy efficiency and conservation (EE&C) programs. Policymakers should also impose mandatory regulations to form energy management teams in industries. Those teams should arrange campaigns/meetings for informing the energy users about EE&C. To ensure competitive pricing and availability for EE products, initiatives need to be taken to develop local entrepreneurship for EE products.

## 5 Conclusion

Energy generation, distribution, and consumption in an efficient way are of prime importance to preserve mankind. This study explores the behavioral factors underlying the energy use pattern in the industrial sector of Bangladesh. Respondents' sociodemographic information indicated that the industrial sector is male dominated and most of the respondents are 20–40 years old. Also, most respondents hold at least an honors degree with an engineering background. The majority of respondents felt that industries pay very little attention to educating, training, being aware, and supervising their employees about energy efficiency and management. Consequentially, most respondents also opined that they are not aware of energy efficient technology. It is found that energy consumption behavior is insensitive to gender and age groups. The sociodemographic variables i.e., level of education and educational background were initially found to be affecting consumption behavior according to the ANOVA test but in the logistic regression model adjustment of other covariates ruled out this effect. The behavioral constructs were found to be initially correlated with personal consumption behavior (PCB) according to Kendal’s tau-b. These correlations were verified in the logistic regression model. According to the logistic regression model, technology adoption norms, training, and supervision, technological ignorance, and responsibility significantly affect PCB.

Exploring the behavioral aspects of energy consumption behavior is a first-of-a-kind study in the industrial sector of Bangladesh. This study covers 20 types of industries with large sample size. There are a few limitations to this study. A sociodemographic variable “income” was not included. Although longitudinal follow-up with interventions can help understand behavioral aspects more precisely, it is not adopted in this study. Also, the assessment of energy-saving behaviors used in this study relies on respondents' self-reports. The validity of the self-report questionnaire method as a means of determining behavioral attitudes is often debated. The study suggests more research on behavioral energy conservation patterns in the industrial sector of Bangladesh.

## Declarations

### Author contribution statement

Islam Md Md Shafiqul: Conceived and designed the experiments; Survey; Analyzed and interpreted the data; Wrote the paper.

Tanvir Hassan Bhuiyan: Performed the experiments, Performed the questionaire design, literature review, colleted survey data and partly analysis data; Contributed reagents, materials, analysis tools or data.

Md Azaharul Islam Rayhan: Conducted statistical analysis, interpreted the data and wrote the paper.

### Funding statement

This work was supported by 10.13039/100009950Ministry of Education, Bangladesh, Grant number SD2017597.

### Data availability statement

Data will be made available on request.

### Declaration of interest’s statement

The authors declare no conflict of interest.

## Additional information

No additional information is available for this paper.

## Appendix A Supplementary data

Questionaire Questionaire
