# Supplementary material for: Behavioral factors underlying energy consumption pattern: A cross-sectional study on industrial sector of Bangladesh
Source: Heliyon. 2022 Nov 16;8(11):e11523. doi: 10.1016/j.heliyon.2022.e11523 (PMC9691912; doi:10.1016/j.heliyon.2022.e11523)
Supplement: Questionaire [file mmc1.pdf]

Survey Questionnaire on Energy Use Pattern and Potential Energy Saving Opportunity in Industrial Sector

Serial No. of the respondent: Date: Place:

(The survey is completely anonymous and no identifying information will be kept about you. This information is being collected for research purposes only)

Please tick (√) or provide your most appropriate response for each and every question.

I. Demographic information

Gender:	O Male		O Female	
Age Group	O 20-30	O 31-40	O 41-50	O 51-65
Level of Education	O S.S.C/Equivalent	O H.S.C/Equivalent	O Honors/Masters/Ph.D.	
Educational background				
O Science	O Engineering	O Humanities	O Business Studies	O Social Science
Designation:		Department:		

II. Questionnaire on Energy Consumption Behavior and Attitude

1. Thinking back over the past six months, how often have you done the following? Please check the box on the following scale that best represents your view:

Statement	Very often	Often	Sometimes	Less often	Never
Switch off lights in a hallway or restroom when not needed.					
Using skylight whenever possible to minimize lighting cost.					
When leaving office, arrange for the last-man-out to check and switch off the power source to all air conditioning, lighting and office equipment.					
Shut off computer monitors when not needed.					
Set computer power to 'save mode' to minimize power.					
Keeping doors and windows closed when AC is on.					
Use shades and blinds to control direct sun through windows in both summer and winter to prevent or encourage heat gain.					
Instructing/encouraging/advocating colleagues to adopt energy saving.					
Wear informal and lighter clothes during summer season.					
Wear formal and additional clothes during winter season.					

2. Please check the box on the following scale that best represents your view.

Statement	Very often	Often	Sometimes	Less often	Never
I talk about saving energy with colleagues in the office.					

I consider energy management responsibilities as a part of my job.					
I attend Regular management meetings to review energy use.					
Awareness campaigns held regularly.					
Actively report about energy wastage and give suggestions (lights on, doors open, steam leaks etc.					
Maintenance schedules include reducing energy wastage.					

3. Please check the box on the following scale that best represents your estimate about the following energy use related statements

Statement	Strongly disagree	Disagree	Neutral	Agree	Strongly agree
Minimize the heat created by lights, machinery or equipment which are left ‘on’ when not required.					
Turn off all unnecessary lights.					
Employ a lower wattage of lighting where possible.					
Use automatic switches for lights, fans and AC to keep them off in the unoccupied spaces.					

III. Questionnaire on Energy Conservation and Efficiency

4. To the best of your knowledge which (if any) of the following apply to you / your organization?

Statement	Strongly oppose	Somewhat oppose	Neutral	Somewhat support	Strongly support
Authority organize training to encourage and support staff in reducing energy consumption.					
Employees are trained properly to interact with any energy management System that exists.					
Behavioral changes in energy use are important in my office					
There should be an energy team to educate employees and operators about energy efficiency.					
I regularly watch documentary program regarding energy consumption issue in the television/internet.					
I read lots of articles regarding energy consumption from the book/Magazine/ Newspaper.					

5. The major barriers for not adopting the energy efficient technology/practice

Statement	Strongly oppose	Somewhat oppose	Neutral	Somewhat support	Strongly support
Not aware of energy efficient technology.					
Energy efficiency products do not always save energy.					
I don’t use it too often.					
Lifecycle of products is too short.					
I know nothing about energy efficiency labeling.					

6. To what extent about energy use do you agree or disagree about yourself

Statement	Strongly disagree	Disagree	Neutral	Agree	Strongly agree
Taking action to reduce energy use is convenient for me.					
My action to reduce energy use is effective.					
In our meetings we discuss about energy efficiency.					
I have moral obligation to reduce my energy usage.					

7. According to your opinion, which is the most important solution for reduction in office use energy consumption

Statements	Very important	Somewhat important	Not important	Not important at all	Not sure
Raising awareness and changing human behaviors.					
Using more energy efficient appliances.					
Making improvements in building designs.					
Opinion/comment					
Do you think we should have an energy culture to reduce energy waste?	○ Yes<br>○ No<br>If yes, please write your comment below.				
Comment:					

We greatly appreciate your kind co-operation.
